# Anti-Obesity Effect of *Artemisia capillaris* Extracts in High-Fat Diet-Induced Obese Rats

**DOI:** 10.3390/molecules18089241

**Published:** 2013-08-02

**Authors:** Dong Wook Lim, Yun Tai Kim, Yu-Jung Jang, Young-Eon Kim, Daeseok Han

**Affiliations:** Functionality Evaluation Research Group, Korea Food Research Institute, Seongnam 463-746, Korea

**Keywords:** *Artemisia capillaris*, scoparone, hypolipidemic, anti-obesity

## Abstract

This study evaluated the anti-obesity effects of *Artemisia capillaris* extracts in high-fat diet (HFD)-induced obese rats. After six weeks feeding with HFD, Wistar male rats (12-weeks-old) were divided into three groups: HFD-control group and HFD mixed with 0.4% and 0.8% *Artemisia capillaris* extracts treated groups. After seven weeks of treatments, the body weight gain of the 0.4% and 0.8% *A. capillaris* extracts treated groups were significantly less than that of the HFD-control group by 11.8% and 15.4%, respectively. Also, *A. capillaris* extracts treated groups showed significantly lower serum TG, TC and LDL-c levels in a dose-related manner, while causing the reverse effect in serum HDL-c, and exhibited a hepatoprotective effects *in vivo*, indicated by reduced hepatic lipid contents, and serum ALT and AST levels. These results show that *A. capillaris* extracts may prevent body weight increases and improve dyslipidemia in HFD-induced obese rats by enhancing their lipid metabolism.

## 1. Introduction

Obesity is often associated with dyslipidemia, cardiovascular risks, hypertension and type 2 diabetes mellitus and has recognized as one of the most serious public health problems [1]. Numerous drugs have been approved for the treatment of obesity; however, most of them have been withdrawn from the market because of their serious adverse effects, leaving only Orlistat, a lipase inhibitor [2]. Therefore, many studies have been conducted to find and develop the new anti-obesity drugs or dietary supplements through the use of medicinal plants that could minimize the side effects [3,4].

The plant genus *Artemisia*, that includes over 1,500 species, has been a rich source of remedies in many countries [5]. *Artemisia capillaris* Thunb (Yin Chen Hao), which belongs to the family of Asteraceae, is one of the earliest and most important edible crude herbs used for various medicinal purposes in East Asian countries, including Korea and China. *A. capillaris* has been widely used as a hepatoprotective, analgesic and antipyretic agent [6]. Many studies have shown various biological activities, such as hypoglycemic [7], hypolipidemic [8,9], anti-inflammatory [10], and anti-carcinogenic [11] effects. *A. capillaris* in particular has shown dramatic hepatoprotective effects in chemical induced liver injury models [12,13,14]. Scoparone, a derivative of coumarin (1,2-benzopyrone) is a major component of *A. capillaris* and has potent hepatoprotective [15], immunosuppressive [16], vascular relaxant [17] and anti-diabetic effects [18]. These reports led us to hypothesize that high content of scoparone in *A. capillaris* extracts could serve as a potent therapeutic agent for the obesity. However, their efficacy needs to be scientifically evaluated *in vivo* experiments.

In the present study, different polarities of solvent were selected as extraction solvents to investigate which solvent is better in extracting scoparone from *A. capillaris*, and their total phenolic content (TPC) and antioxidant activity were evaluated. Then, the anti-obesity effects of the selected *A. capillaris* extracts was further investigated by high-fat diet (HFD)-induced obese rats.

## 2. Results and Discussion

### 2.1. TPC and Antioxidant Activity in Different Extracts of *A. capillaris*

**Figure 1:**
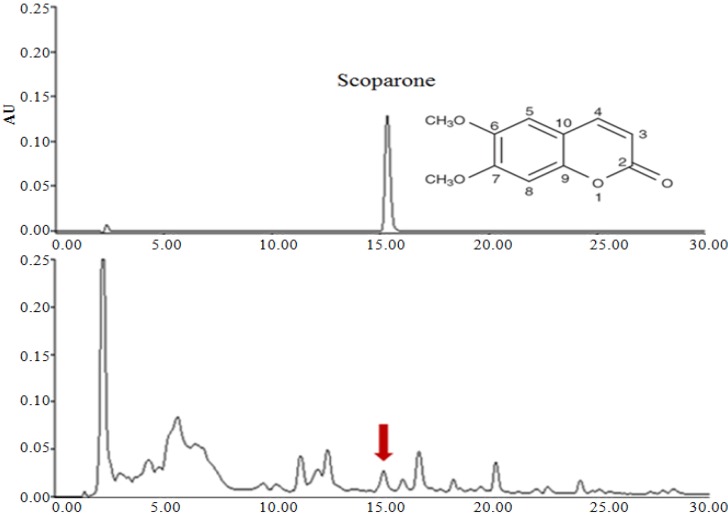
2-D HPLC chromatograms for standardization of *A. capillaris* extracts.

As shown in Table 1, it seems that the best results are obtained by using a more polar solvent (aqueous ethanol, 50%, v/v). Alcoholic solvents (methanol and ethanol) have been commonly used to extract polyphenols from natural sources. Mixtures of alcohols and water have revealed to be more efficient in extracting phenolic compounds compared to mono-component solvents. Addition of small amounts of water to organic solvents creates a more polar medium which facilitates phenolic extraction [19]. The scoparone obtained at 15 min has identical retention time and monitored at 274 nm as a main active compound of *A. capillaris* extracts. This was confirmed by adding a standard of scoparone to the analyzed sample. *A. capillaris* extracts was standardized to contain 2.81 ± 0.21 mg/g scoparone (Figure 1).

**Table 1:** TPC, antioxidant activity and scoparone in different ethanol concentrations.

Ethanol Concentration (%, v/v)	Yield (%)	TPC (mg GAE/g)	RSC (%)	Scoparone (mg/g)
100	10.4	86.8 ± 0.09	61.9 ± 1.00	1.16 ± 0.13
70	15.2	99.8 ± 0.03	63.4 ± 1.49	1.97 ± 0.09
50	15.8	139.2 ± 0.40 ^a^	74.1 ± 0.75 ^a^	2.81 ± 0.21 ^a^

Data are expressed as mean ± SD. ^a^ A significant increase at *p* < 0.05, when compared with 100% ethanol concentration.

### 2.2. Effects of *A. capillaris* Extracts on Body Weight Gain and Food Intake

**Figure 2:**
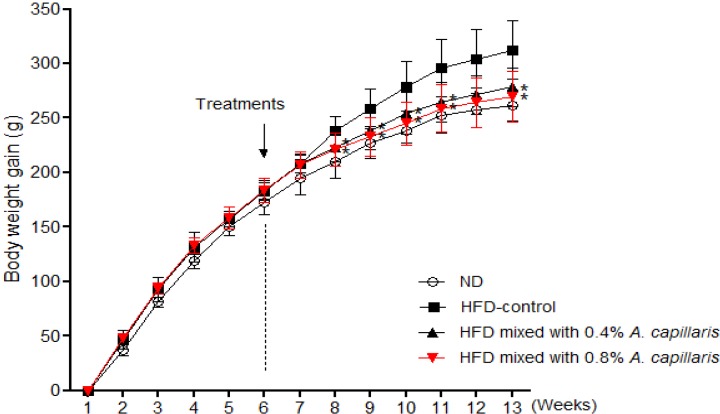
Effects of *A. capillaris* extracts on body weight gain in HFD-induced obese rats. The body weight of the animals was recorded weekly during the experimental period. The body weight gain was calculated by the equation: final body weight − initial body weight. Data are mean ± SD (n = 12 per group). * *p* < 0.05, significant difference from the HFD-control group.

In the six successive weeks feeding of HFD, the body weight gain in the HFD feeding group was significantly higher (169.8 ± 5.1 g *vs.* 183.7 ± 2.3 g, 8.3%, *p* < 0.01) than in the normal diet group. A significant difference in body weight gain was observed between the *A. capillaris* extracts treated group and the HFD-control group by two weeks after initiating treatments (Figure 2). After seven weeks of treatments, the body weight gains of the 0.4% and 0.8% *A. capillaris* extracts treated groups were significantly less than that of the HFD-control group by 11.8% and 15.4%, respectively (Table 2). No abnormal clinical signs were observed during the experimental periods. Also, there were no significant differences among the HFD feeding groups in food intake.

**Table 2:** Effects of *A. capillaris* extracts on body weight changes, epididymal adipose tissue weight, and food intake in HFD-induced obese rats.

Groups	Body weight (g)		Body weight gain (g/day)	Food intake (g/day)	Energy intake (kcal/day)	Epididymal adipose tissue (g)
	Initial	Final				
I	181.4 ± 8.7	439.6 ± 20.7	3.1 ± 0.2	17.4 ± 0.3	64.1 ± 1.5	9.8 ± 3.1
II	181.3 ± 7.5	498.7 ± 29.1	3.7 ± 0.3	16.8 ± 0.1	87.8 ± 2.1	15.1 ± 2.2
III	180.2 ± 7.2	458.6 ± 16.1 ^a^	3.3 ± 0.2	16.6 ± 0.2	88.7 ± 0.8	12.8 ± 4.8 ^a^
IV	179.7 ± 5.4	449.2 ± 26.2 ^b^	3.2 ± 0.3 ^a^	16.6 ± 0.1	87.4 ± 0.6	11.2 ± 2.5 ^b^

Group I = normal diets (ND); Group II = high fat diet (HFD)-control; Group III = HFD mixed with 0.4% *A. capillaris*; Group IV = HFD mixed with 0.8% *A. capillaris*. Data are expressed as mean ± SD. ^a^ A significant decrease at *p* < 0.05, when compared with Group II values. ^b^ A significant decrease at *p* < 0.01, when compared with Group II values.

### 2.3. Effects of A. capillaris Extracts on Serum Lipoproteins Levels

Serum TG, TC and LDL-c levels were significantly higher in the HFD-control group compared with the normal diet group. After seven weeks of treatments, 0.4% and 0.8% *A. capillaris* extracts treated groups showed significantly lower serum TG, TC and LDL-c levels in a dose-related manner while causing the reverse on serum HDL-c. Similarly, *A. capillaris* extracts caused significant reductions in the atherogenic and coronary artery risk indices (Table 3).

**Table 3:** Effects of *A. capillaris* extracts on serum lipoproteins in HFD-induced obese rats.

Groups	TC (mg/dL)	TG (mg/dL)	HDL-c (mg/dL)	LDL-c (mg/dL)	CRI	AI
I	77.7 ± 10.5	77.7 ± 6.1	45.5 ± 4.1	16.7 ± 8.1	1.7 ± 0.2	0.4 ± 0.2
II	94.8 ± 6.1 ^c^	98.3 ± 12.4 ^c^	44.5 ± 4.3	33.6 ± 4.2 ^c^	2.3 ± 0.3 ^c^	0.8 ± 0.2 ^c^
III	83.0 ± 8.9 ^a^	87.7 ± 9.7 ^a^	48.2 ± 4.8	17.3 ± 5.4 ^a^	1.7 ± 0.1 ^a^	0.4 ± 0.1 ^b^
IV	82.0 ± 6.6 ^a^	81.9 ± 9.6 ^a^	47.9 ± 4.6	17.9 ± 4.8 ^a^	1.7 ± 0.2 ^a^	0.4 ± 0.3 ^b^

Data are mean ± SD (n = 12 per group). ^a^ A significant decrease at *p* < 0.05, when compared with Group II values. ^b^ A significant decrease at *p* < 0.01, when compared with Group II values. ^c^ A significant increase at *p* < 0.05, when compared with Group I values. CRI: coronary artery risk index, AI: atherogenic index.

### 2.4. Effects of *A. capillaris* Extracts on HFD-Induced Fatty Liver

**Figure 3:**
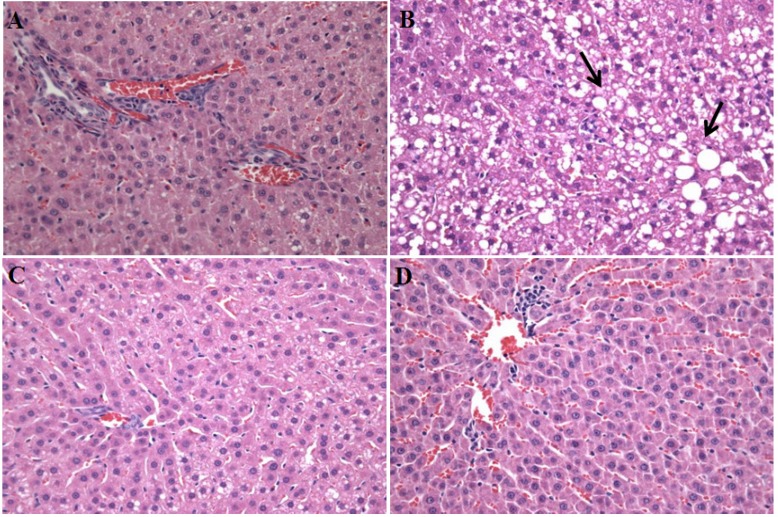
Histological images of liver tissues from HFD-induced obese rats hepatic steatosis displaying the hepatoprotective effects of *A. capillaris* extracts. There were no histological abnormalities observed in normal diets rats (**A**). Rats fed with a HFD for 13 weeks develop a high degree of steatosis, showing hepatocytes with severe cytoplasmic vacuoles and swelling (**B**). The treatments with 0.4% and 0.8% *A. capillaris* extracts resulted in the prevention of hepatic fatty deposition in hepatocytes (**C**,**D**). The tissues were surgically excised and subjected to histological study by staining with hematoxylin and eosin (H&E). All magnifications: 100×. The arrows indicate fatty hepatocytes.

As shown in Table 4, *A. capillaris* extracts exhibits a hepatoprotective effect *in vivo*, indicated with reduced hepatic lipid contents, and serum ALT and AST levels. It was found that rats fed with HFD alone developed a high degree of steatosis, with severe cytoplasmic vacuoles and hepatocyte swelling (Figure 3B), whereas no histological abnormalities were observed in normal control rats (Figure 3A). The effect of increased liver enzymes levels and the formation of steatosis in the HFD-control group correlate with a significant increase of liver weight. The treatments of *A. capillaris* extracts resulted in the prevention of hepatic fatty deposition in hepatocytes (Figure 3C,D).

**Table 4:** Effects of *A. capillaris* extracts on HFD-induced fatty liver.

Groups	Liver weight (g)	SOD (U/mg protein)	Serum (mg/dL)		Liver lipid (mg/g wet wt)	
			AST	ALT	TC	TG
I	10.0 ± 0.6	2.42 ± 0.5	115.1 ± 11.6	62.1 ± 6.9	6.1 ± 0.7	37.2 ± 4.9
II	13.7 ± 0.8 ^c^	1.98 ± 0.7	144.4 ± 25.7 ^c^	84.5 ± 6.6 ^c^	10.1 ± 0.8 ^c^	64.8 ± 7.4 ^c^
III	10.7 ± 0.8 ^a^	2.41 ± 0.6	123.1 ± 16.4 ^a^	70.3 ± 4.9 ^b^	7.2 ± 0.6 ^a^	52.8 ± 6.8 ^a^
IV	10.2 ± 0.7 ^a^	2.42 ± 0.5	118.6 ± 10.5 ^b^	67.5 ± 6.8 ^b^	6.5 ± 0.5 ^a^	44.1 ± 5.8 ^a^

Data are mean ± SD (n = 12 per group). ^a^ A significant decrease at *p* < 0.05, when compared with Group II values. ^b^ A significant decrease at *p* < 0.01, when compared with Group II values. ^c^ A significant increase at *p* < 0.05, when compared with Group I values.

### 2.5. Discussion

In the present study, the rats fed the HFD for thirteen weeks showed obesity, which was associated with significantly increased body weight and the development of dyslipidemia. After seven weeks treatment with an *A. capillaris* extracts, the body weight gain and serum lipoproteins levels were significantly lower than those of the HFD-control group, without affecting the amount of food intakes. Although measurement of food consumption was relatively crude, the results of the study suggest that chronic treatments of *A. capillaris* extracts might have a hypolipidemic effects in HFD-induced obese rats without affecting appetite. This suggestion was supported by scoparone from *A. capillaris*, which inhibits lipid peroxidation and protection against the alterations of serum lipoproteins in hyperlipidemic diabetic rabbits [20,21].

Obesity is characterized by increased adipose tissue mass that results from both increased fat cell number and increased fat cell size. Adipose tissue is a dynamic organ that plays an important role in energy balance and changes in mass according to the metabolic requirements of the organism [22]. Excess energy intake and reduced energy expenditure results in abnormal excessive growth of white adipose tissue (WAT), which can lead to the development of obesity [23]. Epididymal adipose tissue in the rat is generally considered to be WAT with a characteristic structure and function [24]. In our results, the weight of epididymal adipose tissue in *A. capillaris* extracts treated group was significantly decreased compared to the HFD-control group. These results suggest that *A. capillaris* extracts may prevent the accumulation of WAT in HFD-induced obese rats. However, further mechanism studies are needs to clarify that the anti-obesity effects of *A. capillaris* extracts may be elicited by regulating the expressions of lipogenesis-related genes in WAT.

The effect of *A. capillaris* extracts treatment on the atherogenic and coronary artery risk indices is also notable. The ratio of total cholesterol to HDL-c (atherogenic index) and the ratio of LDLc to HDL-c (coronary artery index) are strong and reliable indicators of whether or not cholesterol is deposited into tissues or metabolized and excreted [25]. In humans, the normal reference values for atherogenic index and coronary artery index should not be higher than 4 and 2.5, respectively. Thus, patients with cardiac risk indices higher than these reference values are predisposed to developing ischaemic heart disease and thrombotic cardiovascular accident [26]. In this present study, results showed that treatment with *A. capillaris* extracts caused profound reductions in the atherogenic and coronary indices in HFD-induced obese rats, and these strongly suggest that *A. capillaris* extracts possesses cardioprotective potential.

We also analyzed the effects of *A. capillaris* extracts on the development of fatty liver, which is strongly associated with obesity [27]. Defects in fat metabolism are responsible for the pathogenesis of hepatic steatosis, which may be due to an imbalance in energy consumption and its combustion, resulting in lipid storage [28]. Also, it is noteworthy that diminished SOD represents important components of the antioxidant defense enzyme [29], and increased systemic oxidative stress has been found in HFD-induced obese in vivo model [30]. Moreover, Xu *et al.* reported that the liver of the HFD-induced obese rodents exhibited an accumulation of numerous fatty droplets; a typical sign of fatty liver, and the liver weight was significantly higher in the HFD-control group than in the normal diet group [31,32]. Our results indicated that the continuous consumption of HFD may play a role in the development of hepatic steatosis associated with obesity, and *A. capillaris* extracts exhibits a hepatoprotective effect, indicated with improved liver weight, hepatic lipid contents and SOD activity. Serum AST and ALT levels are clinically and toxicologically important indicators [33], and increase as a results of tissue damage caused by toxicants or disease conditions. In the HFD-control group, the activities of liver function markers, including serum AST and ALT, were significantly elevated relative to those in the normal diet group and were improved by *A. capillaris* extracts supplementation. These results suggest that *A. capillaris* extracts supplementation may attenuate the development of hepatic steatosis and that *A. capillaris* extracts is potentially effective in ameliorating fatty liver in HFD-induced obese rats.

## 3. Experimental

### 3.1. Determination of Scoparone in *A. capillaris* Extracts

*A.* capillaris was collected in Kang Won Do, Korea. The sample was identified by Dr. Young-Eon Kim and Voucher specimen was deposited in the Functionality Evaluation Research Group, Korea Food Research Institute, Seongnam, Korea. *A. capillaris* was extracted with three different solvents (50, 70 and 100% ethanol) using an ultrasonic cleaning bath (model 5510, Branson, Danbury, CT, USA) for 12 h. Then each extracts were centrifuged at 10,000 g for 30 min at 25 °C. Supernatants were filtered through a membrane filter (0.45 µm; Millipore, Billerica, MA, USA). After removing the solvents via rotary evaporation, the remaining extracts were vacuum dried to an average yield of about 8.7% (w/w). The quantitative authentication of scoparone was performed by a high performance liquid chromatography (HPLC) analysis system equipped with a Waters 1525 pump, a 2707 auto sampler and a 2998 PDA detector. The chromatic separation was achieved at 30 °C on Waters Sunfire™ C18 (250 mm × 4 mm i.d., 5 μm particle size) column. *A. capillaris* extracts was monitored at 274 nm for scoparone. The mobile phase was acetonitrile and water (25:75, v/v). The run time was set at 30 min, and the flow rate was 1.0 mL/min (injection volume of 10 μL). Then, the scoparone was isolated at the retention time of 15.42 min

### 3.2. TPC and Antioxidant Activity in *A. capillaris* Extracts

TPC of the *A. capillaris* extracts was determined by the Folin-Denis method [34]. The estimation of phenolic compounds in the extracts was carried out in five replicates and calculated by a calibration curve obtained with gallic acid. TPC was expressed as gallic acid equivalents (mg GAE/g dry matter). Antioxidant activity of *A. capillaris* extracts was determined using DPPH method [35]. Briefly, *A. capillaris* extracts was dissolved in methanol. Different volumes (50, 100 and 150 μL) of the extracts were mixed in test tubes with 1,000 μL of DPPH solution (0.1 mM in methanol) a made up to final volume of 4 mL with methanol. The mixture was shaken vigorously and allowed to stand at room temperature for 30 min. Then the absorbance was measured at 517 nm against methanol as the blank in a spectrophotometer. The experiment was performed in triplicate and the average absorption was noted for each concentration. Radical scavenger capacity (RSC%) was calculated using the following formula: RSC% = (absorbance of control absorbance of sample)/ absorbance of control × 100.

### 3.3. Animals and Diets

Five-week-old male Wistar rats (Japan SLC, Inc., Hamamatsu, Japan), were housed at two rats per cage in an air-conditioned room at 23 ± 1 °C, 55%–60% relative humidity, and a 12 h light/dark cycle (lights on at 07:00 and lights off at 19:00), and were given a laboratory regular rodent diet; normal diet (AIN-93, Research Diets Inc., New Brunswick, NJ, USA) for one week to adapt to their environment before the experiments. A purified ingredient HFD with 40 Kcal% fat primarily from lard (D12492, Research Diets Inc.) was used to induce a rapid increase in body weight and obesity. After six weeks feeding with HFD, the animals were randomly divided into four groups (n = 12) in each group such that the weight difference within and between groups does not exceed ±20% of the average body weight of the sample population. The composition of the diet for each experimental group is shown in Table 5. The rats had free access to food and water, and their food consumption was measured daily while their body weight was measured weekly for seven weeks, starting at twelve-week-old. At the end of the feeding period, the rats were fasted for 12 h. The blood was collected from the abdominal aorta, and their livers and epididymal adipose tissues were collected for the experiments described later in this section. All animal experiments were carried out according to the guidelines of the Korea Food Research Institutional Animal Care and Use Committee.

**Table 5:** Composition of the experimental diets.

Ingredients	Experimental Groups			
	ND	HFD	HFD mixed with *A. capillaris*	
			0.40%	0.80%
Casein	20	20	20	20
Sucrose	10	10	10	10
Corn starch	39.75	24.75	24.35	23.95
Soybean oil	7	7	7	7
Dextrose	13.2	13.2	13.2	13.2
Lard	-	15	15	15
Cellulose	5	5	5	5
L-cystine	0.3	0.3	0.3	0.3
AIN-mineral mixture	3.5	3.5	3.5	3.5
AIN-vitamin mixture	1	1	1	1
Choline bitrate	0.25	0.25	0.25	0.25
*A. capillaris* extracts	-	-	0.4	0.8
Total (%)	100	100	100	100
Energy (Kcal/100 g)	380.2	487	487	487

### 3.4. Biochemical Parameter Analysis

The serum samples were prepared by centrifugation of the collected blood samples (1,013 g for 15 min at 4 °C), then stored at −80 °C for biochemical determinations. The serum concentrations of total cholesterol (TC), triglyceride (TG), high density lipoprotein-cholesterol (HDL-c), alanine transaminase (ALT), and aspartate aminotransferase (AST) were determined using an automatic analyzer (ADVIA 1650, Bayer, Tokyo, Japan). Serum LDL-c was estimated using Frieldwann's equation: LDL-c = [TC-(HDL-c + TG/5)]. Atherogenic index (AI) and coronary risk index (CRI) were calculated as: LDL-c/HDL-c [36] and TC /HDL-c [37]. The hepatic lipid was extracted using the procedure developed by Folch *et al* [38], and TC and TG contents were determined using a commercial enzymatic kit (Asan Pharm. Co., Seoul, Korea). One part of the liver tissue was washed and homogenized (1:10, w/v) in ice-cold 50 mM Tris buffer (pH = 7.4). The contents were centrifuged at 10,000 g for 20 min at 4 °C, and the supernatant obtained was analyzed for superoxide dismutase (SOD) [39].

### 3.5. Liver Histological Analysis

The liver tissues were divided into the Tissue Freezing Medium (TBS, Durham, NC, USA) and then deep-frozen in liquid nitrogen for their preservation. The liver tissues were cut into 5 μm thick sections using a cryostat instrument (CM 1850; Leica, Heidelberg, Germany). The tissue sections were fixed in 10% neutral phosphate-buffered formalin solution. The lipids and nuclei of the liver cells were stained with hematoxylin and eosin (H&E). A diagnosis of fatty liver was made based on the presence of macro- or microvesicular fat in >5% of the hepatocytes in a given slide.

### 3.6. Statistical Analysis

All data were presented as the mean ± standard deviation (SD). The effects of different treatments were compared by one-way ANOVA test, followed by the post-hoc Tukey_test for multiple comparisons using GraphPad Prism 5 (GraphPad Software Inc., La Jolla, CA, USA). *p* < 0.05 was considered statistically significant.

## 4. Conclusions

In conclusion, *A. capillaris* extracts may prevent body weight increases and improve dyslipidemia in HFD-induced obese rats by enhancing lipid metabolism.
